# Twisted space-frequency and space-time partially coherent beams

**DOI:** 10.1038/s41598-020-68705-9

**Published:** 2020-07-24

**Authors:** Milo W. Hyde

**Affiliations:** Air Force Institute of Technology, Dayton, OH 45433 USA

**Keywords:** Engineering, Optics and photonics, Physics

## Abstract

We present partially coherent sources that are statistically twisted in the space-frequency and space-time domains. Beginning with the superposition rule for genuine partially coherent sources, we derive source plane expressions for the cross-spectral density (CSD) and mutual coherence functions (MCFs) for twisted space-frequency and space-time Gaussian Schell-model (GSM) beams. Using the Fresnel approximation to the free-space Green’s function, we then paraxially propagate the CSD and MCF to any plane $z > 0$. We discuss the beams’ behavior as they propagate, with particular emphasis on how the beam shape rotates or tumbles versus *z*. To validate our analysis, we simulate the generation and subsequent propagation of twisted space-frequency and space-time GSM beams. We compare the simulated moments to the corresponding theoretical predictions and find them to be in excellent agreement. Lastly, we describe how to physically synthesize twisted space-frequency and space-time partially coherent sources.

## Introduction

Approximately 30 years ago, Allen et al. discovered that fields with a vortex wavefront, e.g., Laguerre–Gaussian beams, carried orbital angular momentum (OAM)^1,2^. This initial discovery has spawned much research in applying vortex beams in free-space optical communications, optical tweezers, astronomy, etcetera^3–8^.

In 1993, Simon and Mukunda introduced the concept of a twisted partially coherent field^9^. Like fields with vortex phase fronts, twisted partially coherent beams carry OAM. They differ from traditional vortex fields in that the twist, or rotation is statistical in nature and exists only in the context of stochastic beams, i.e., the twist disappears in the coherent limit. Like Allen et al.’s seminal OAM work, Simon and Mukunda’s twisted partially coherent beams have generated significant interest in the intervening years^10–21^.

A vast majority of the references pertaining to vortex or twisted beams, whether they be fully coherent or stochastic, deal with spatial vortices or twists in the plane orthogonal to the direction of propagation, e.g., the *x*–*y* plane for a *z* propagating wave. All of these beams possess OAM orientated in the longitudinal direction. Recently, a class of vortex beams has been introduced where the vortex exists in the space-time domain^22–27^. Referred to as spatiotemporal optical vortices (STOVs), these beams carry transverse OAM. Their study and subsequent synthesis opens up the possibility for novel uses in optical manipulation, optical tweezing, and other applications.

Motivated by the existence of STOVs and their potential applications, we introduce the space-frequency and space-time extensions of Simon and Mukunda’s spatially twisted partially coherent beams. Analogous to the relationship between spatial and spatiotemporal vortices, twisted space-frequency and space-time partially coherent sources possess statistical twists between their spatial and temporal dimensions. Like STOVs, these beams carry transverse OAM, and, in addition, rotate or tumble as they propagate.

We emphasize two foundational papers that describe spatiotemporal coupling which are germane to our work. The first is a paper by Akturk et al. that develops a general mathematical theory for spatiotemporal coupling of coherent Gaussian pulsed beams^28^. The second is authored by Wang et al. and presents a 6 × 6 matrix formalism which describes the behavior of partially coherent Gaussian Schell-model (GSM) pulsed beams in linear, dispersive media^29^. Although spatiotemporal coupling has been researched in the past by these and numerous other authors, no one, to our knowledge, has formally presented—mathematically and physically described the propagation behaviors, or generated random field realizations of—twisted space-frequency and space-time partially coherent sources, as we do here.

In the next section, we derive expressions for the cross-spectral density (CSD) and mutual coherence functions (MCFs) for twisted space-frequency and space-time partially coherent beams, respectively. Assuming sources of GSM form^30,31^, we study the behaviors of twisted space-frequency and space-time beams as they propagate in free space by evaluating the CSD and MCF paraxial propagation integrals. We simulate the synthesis and propagation of these beams, and compare the simulated, or sample statistical moments to their corresponding theoretical expressions to validate our analysis. We conclude with a discussion of how to physically synthesize these new partially coherent beams and a summary of our work.

## Methods

In this section, we theoretically introduce twisted space-frequency and space-time partially coherent sources.

### Twisted space-frequency partially coherent sources

Our analysis begins with the necessary and sufficient criterion for a genuine CSD function^32,33^:

1 $$\begin{aligned} W\left( x_1,x_2,\omega _1,\omega _2\right) = \iint _{-\infty }^{\infty }p\left( v_x,v_\omega \right) H\left( x_1, \omega _1; v_x,v_\omega \right) H^*\left( x_2, \omega _2; v_x,v_\omega \right) \text {d}v_x\text {d}v_\omega , \end{aligned}$$

where $\omega$ is the radian frequency, *p* is any positive function, and *H* is an arbitrary kernel. Equation (1) is also referred to as the superposition rule in the literature. For simplicity, we restrict our analysis to one spatial dimension *x*.

Adapting Mei and Korotkova’s^14^ twist kernel, we let *H* be

2 $$\begin{aligned} H\left( x,\omega ;v_x,v_\omega \right)= & {} H\left( x,\omega ;v_x\right) H\left( x,\omega ;v_\omega \right) \nonumber \\ H\left( x,\omega ;v_x\right)= & {} \exp \left( -\frac{\sigma _x}{2} x^2\right) \exp \left( -\frac{\sigma _\omega }{2} {\bar{\omega }}^2\right) \exp \left[ -\text {j}\left( x - \text {j}\alpha \mu \omega \right) v_x\right] \nonumber \\ H\left( x,\omega ;v_\omega \right)= & {} \exp \left( -\frac{\sigma _x}{2} x^2\right) \exp \left( -\frac{\sigma _\omega }{2} {\bar{\omega }}^2\right) \exp \left[ -\text {j}\left( \omega - \text {j}\beta \mu x\right) v_\omega \right] , \end{aligned}$$

where ${\bar{\omega }} = \omega - \omega _c$ and $\omega _c$ is the radian frequency of the light, or carrier wave. We will discuss $\sigma _x$, $\sigma _\omega$, $\alpha$, $\beta$, and $\mu$ later on in the paper. We note that other twist kernels exist in the literature^10,15,18,19,21^ and can be adapted in a similar manner as above to produce twisted space-frequency and space-time beams.

To generate twisted GSM beams, we choose *p* to be^10,14,15,18,19,21^

3 $$\begin{aligned} p\left( v_x,v_\omega \right) = p\left( v_x\right) p\left( v_\omega \right) = \sqrt{\frac{\alpha }{\pi }}\exp \left( -\alpha v_x^2\right) \sqrt{\frac{\beta }{\pi }}\exp \left( -\beta v_\omega ^2\right) . \end{aligned}$$

Like *H* in Eq. (2), other *p* can be used, e.g., the multi-Gaussian *p* in Refs.^14,34^. Substituting Eqs. (2) and (3) into (1) and evaluating the integrals yields a CSD of the form

4 $$\begin{aligned} W\left( x_1,x_2,\omega _1,\omega _2\right)= & {} \exp \left( -\frac{x_1^2+x_2^2}{4W_x^2}\right) \exp \left[ -\frac{\left( x_1-x_2\right) ^2}{2\delta _x^2}\right] \nonumber \\& \times \exp \left( -\frac{{\bar{\omega }}_1^2+{\bar{\omega }}_2^2}{4W_\omega ^2}\right) \exp \left[ -\frac{\left( {\bar{\omega }}_1-{\bar{\omega }}_2\right) ^2}{2\delta _\omega ^2}\right] \exp \left[ \text {j}\mu \left( x_1 {\bar{\omega }}_2 - x_2 {\bar{\omega }}_1 \right) \right] , \end{aligned}$$

where $W_x$ and $W_\omega$ are the beam and spectral pulse widths, $\delta _x$ and $\delta _\omega$ are the spatial coherence and spectral coherence widths, and $\mu$ is the twist parameter. These beam parameters are not independent and are linked in a complex, nonlinear way. Referring back to Eq. (2),

5 $$\begin{aligned} \dfrac{1}{4W_x^2}&= \sigma _x - \dfrac{\beta \mu ^2}{2}\qquad \dfrac{1}{4W_\omega ^2} = \sigma _\omega - \dfrac{\alpha \mu ^2}{2} \nonumber \\ \dfrac{1}{2 \delta _x^2}&= \dfrac{1}{4\alpha } + \dfrac{\beta \mu ^2}{4} \qquad \dfrac{1}{2 \delta _\omega ^2} = \dfrac{1}{4\beta } + \dfrac{\alpha \mu ^2}{4} . \end{aligned}$$

In addition, $\left| \mu \right| \delta _x \delta _\omega \le 1$^31,35,36^, and therefore, the space-frequency twist necessarily disappears in the coherent limit $\delta _x, \delta _\omega \rightarrow \infty$.

Equation (4) has the same basic form as a spatially twisted GSM beam^9,10,12,14,37^; however, here, space and frequency are statistically twisted. It is well known that the spectral density of a spatially twisted stochastic source rotates as the beam propagates. This rotation is in the plane orthogonal to the propagation direction, e.g., *x*–*y* plane for a *z* propagating wave. Twisted space-frequency beams also rotate—this time, in the *x*–$\omega$ plane.

The paraxial, twisted space-frequency GSM CSD at any propagation plane $z > 0$ can be found using the two-frequency Fresnel integral, namely,

6 $$\begin{aligned} W\left( x_1,x_2,\omega _1,\omega _2,z\right)&=  \frac{\exp \left[ \text {j}\left( k_1-k_2\right) z\right] \exp \left[ \frac{\text {j}}{2z}\left( k_1 x_2^2-k_2 x_2^2\right) \right] }{z\sqrt{\lambda _1 \lambda _2}} \nonumber \\&\quad \times \iint _{-\infty }^{\infty } W\left( x_1^{'},x_2^{'},\omega _1,\omega _2\right) \exp \left[ \frac{\text {j}}{2z}\left( k_1 x^{'}_{2}{^{2}}-k_2 x^{'}_{2}{^2}\right) \right] \nonumber \\&\quad\times \exp \left[ -\frac{\text {j}}{z}\left( k_1 x_1 x_1^{'} - k_2 x_2 x_2^{'}\right) \right] \text {d}x_1^{'} \text {d}x_2^{'}, \end{aligned}$$

where $k_{1,2} = \omega _{1,2}/c$ and $\lambda _{1,2} = 2\pi /k_{1,2}$ are the wavenumbers and wavelengths associated with $\omega _1$ and $\omega _2$, respectively and *c* is the speed of light. Substituting in Eq. (4) and after much calculus and algebra,

7 $$\begin{aligned} W\left( x_1,x_2,\omega _1,\omega _2,z\right)= & {} \frac{\sqrt{k_1 k_2}}{2 z \Omega } \exp \left[ \text {j}\left( k_1-k_2\right) z\right] \exp \left( -\frac{{\bar{\omega }}_1^2+{\bar{\omega }}_2^2}{4W_\omega ^2}\right) \exp \left[ -\frac{\left( {\bar{\omega }}_1-{\bar{\omega }}_2\right) ^2}{2\delta _\omega ^2}\right] \nonumber \\&\times \exp \left[ \frac{\text {j} \mu ^2}{8 z \Omega ^2}\left( k_1 {\bar{\omega }}_1^2 - k_2 {\bar{\omega }}_2^2 \right) \right] \exp \left[ \frac{\text {j}}{{2z}} \left( 1 - \frac{k_1 k_2}{4 z^2 \Omega ^2}\right) \left( k_1 x_1^2 - k_2 x_2^2\right) \right] \nonumber \\& \nonumber \\&\times \exp \left[ -\frac{\left( \mu {\bar{\omega }}_1 - k_2 x_2/z \right) ^2 + \left( \mu {\bar{\omega }}_2 - k_1 x_1/z \right) ^2}{16 W_x^2 \Omega ^2} \right] \nonumber \\& \times \exp \left\{ - \frac{\left[ \left( \mu {\bar{\omega }}_1 - k_2 x_2/z \right) - \left( \mu {\bar{\omega }}_2 - k_1 x_1/z \right) \right] ^2}{8 \delta _x^2 \Omega ^2}\right\} \nonumber \\&\times \exp \left[ \text {j} \mu \frac{k_1 k_2}{4 z^2 \Omega ^2} \left( x_1 {\bar{\omega }}_2 - x_2 {\bar{\omega }}_1 \right) \right] , \end{aligned}$$

where $$\Omega ^2 = \left[ \sigma - \text {j}k_1/\left( 2z\right) \right] \left[ \sigma + \text {j}k_2/\left( 2z\right) \right] - \left[ 1/\left( 2\delta _x^2\right) \right] ^2$$ and $$\sigma = 1/\left( 4W_x^2\right) + 1/\left( 2\delta _x^2\right) $$.

The exponentials on the third, fourth, and fifth lines of Eq. (7) correspond to the beam shape, coherence, and twist, respectively. Because of the initial space-frequency coupling, the spectral content of the beam affects its spatial distribution and equivalently, vice versa.

The spectral density *S* of the source can be found by evaluating Eq. (7) at the same space and frequency points^30,31^, i.e.,

8 $$\begin{aligned} S\left( x,\omega ,z\right)= & {} W\left( x,x,\omega ,\omega ,z\right) \nonumber \\= & {} \frac{N_F}{\sqrt{1+4\gamma _x^2+N_F^2}} \exp \left[ -\left( 1 + \mu ^2\frac{4W_x^2 W_\omega ^2}{1+4\gamma _x^2+N_F^2}\right) \frac{{\bar{\omega }}^2}{2W_\omega ^2}\right] \nonumber \\& \times \exp \left[ -\left( \frac{N_F^2}{1+4\gamma _x^2+N_F^2}\right) \frac{x^2}{2W_x^2}\right] \exp \left( \mu \frac{2 N_F}{1+4\gamma _x^2+N_F^2}{\bar{\omega }}x\right) , \end{aligned}$$

where $\gamma _x^2 = W_x^2/\delta _x^2$ and $N_F = 2 k W_x^2/z$ is the coherent Gaussian beam Fresnel number. In order, the exponentials in Eq. (8) physically correspond to the spectral beam shape, spatial beam shape, and *x*-$\omega$ plane rotation. We note that the coefficient in the spectral beam shape exponential is greater than or equal to one. This, when combined with the fact that the spatial beam shape is only affected by diffraction (depends on Fresnel number, spatial beam size, and coherence radius), means that the beam essentially “trades” spectrum to rotate. As $z \rightarrow \infty$ or $N_F \rightarrow 0$, the spectral beam radius asymptotes (the spectral beam shape does not appreciably change), diffraction dominates, and the beam no longer rotates.

Although evident from the numerous references describing rotating coherent beams^28,37–39^, it is important to point out that beam rotation does not imply partial coherence. Rotation, therefore, is a characteristic of partially coherent twisted beams, not a defining characteristic.

### Twisted space-time partially coherent sources

Similar to the approach we used above to produce twisted space-frequency sources, we can also construct twisted space-time partially coherence sources. In many respects, these sources are more physically intuitive than their twisted space-frequency counterparts, as the rotation occurs in the *x*–*t* plane. Paraxially, *t* is closely related to the propagation direction $z$, and therefore, these beams rotate or tumble as the beam propagates.

Like above, we begin with the superposition rule, this time for the MCF $\Gamma$^32,33^:

9 $$\begin{aligned} \Gamma \left( x_1,x_2,t_1,t_2\right)= & {} \int _{-\infty }^{\infty }p\left( v_x\right) H\left( x_1,t_1;v_x\right) H^*\left( x_2,t_2;v_x\right) \text {d}v_x \int _{-\infty }^{\infty }p\left( v_t\right) H\left( x_1,t_1;v_t\right) H^*\left( x_2,t_2;v_t\right) \text {d}v_t , \end{aligned}$$

where *p* and *H* are

10 $$\begin{aligned} p\left( v_x,v_t\right)&= p\left( v_x\right) p\left( v_t\right) = \sqrt{\frac{\alpha }{\pi }}\exp \left( -\alpha v_x^2\right) \sqrt{\frac{\beta }{\pi }}\exp \left( -\beta v_t^2\right) \nonumber \\ H\left( x,t;v_x,v_t\right)&=  H\left( x,t;v_x\right) H\left( x,t;v_t\right) \nonumber \\ H\left( x,t;v_x\right)&= \exp \left( -\frac{\sigma _x}{2} x^2\right) \exp \left( -\frac{\sigma _t}{2} t^2\right) \exp \left( -\text {j}\frac{\omega _c}{2} t\right) \exp \left[ -\text {j}\left( x - \text {j}\alpha \mu t\right) v_x\right] \nonumber \\ H\left( x,t;v_t\right)&= \exp \left( -\frac{\sigma _x}{2} x^2\right) \exp \left( -\frac{\sigma _t}{2}t^2\right) \exp \left( -\text {j}\frac{\omega _c}{2} t\right) \exp \left[ -\text {j}\left( t - \text {j}\beta \mu x\right) v_t\right] . \end{aligned}$$

Substituting the above *p* and *H* into Eq. (9) and evaluating the integrals produces an MCF of the form

11 $$\begin{aligned} \Gamma \left( x_1,x_2,t_1,t_2\right)= & {} \exp \left( -\frac{x_1^2+x_2^2}{4W_x^2}\right) \exp \left[ -\frac{\left( x_1-x_2\right) ^2}{2\delta _x^2}\right] \exp \left[ -\text {j}\omega _c\left( t_1-t_2\right) \right] \nonumber \\&\quad \times \exp \left( -\frac{t_1^2+t_2^2}{4W_t^2} \right) \exp \left[ -\frac{\left( t_1-t_2\right) ^2}{2\delta _t^2}\right] \exp \left[ \text {j}\mu \left( x_1 t_2- x_2 t_1\right) \right] . \end{aligned}$$

The physical source parameters $W_x$, $W_t$, $\delta _x$, $\delta _t$, and $\mu$ are related to $\sigma _x$, $\sigma _t$, $\alpha$, and $\beta$ in the same way as the corresponding twisted space-frequency beam parameters—see Eq. (5). As expected, when $\mu = 0$, Eq. (11) simplifies to a traditional GSM pulsed beam^29,40–44^.

We can propagate the MCF in Eq. (11) to any plane $z > 0$ using the following integral expression:

12 $$\begin{aligned} \Gamma \left( x_1,x_2,t_1,t_2,z\right)= & {} \frac{1}{\lambda _c z} \iint _{-\infty }^{\infty } \Gamma \left[ x_1',x_2',t_1-\frac{z}{c}-\frac{\left( x_1-x_1'\right) ^2}{2cz},t_2-\frac{z}{c}-\frac{\left( x_2-x_2'\right) ^2}{2cz}\right] \text {d}x_1'\text {d}x_2'. \end{aligned}$$

This relation is accurate in the paraxial regime, and if the source is narrowband, i.e., $\omega _c \gg \max \left( 1/W_t,1/\delta _t\right)$. Substituting Eqs. (11) into (12) and neglecting terms greater than second order produces

13 $$\begin{aligned} \Gamma \left( x_1,x_2,t_1,t_2,z\right)\approx & {} \frac{k_c}{2z\Omega } \exp \left[ -\text {j}\omega _c \left( {\bar{t}}_1-{\bar{t}}_2\right) \right] \exp \left( -\frac{{\bar{t}}_1^2 + {\bar{t}}_2^2}{4W_t^2}\right) \exp \left[ -\frac{\left( {\bar{t}}_1 - {\bar{t}}_2 \right) ^2}{2\delta _t^2} \right] \nonumber \\& \times \exp \left[ \frac{\text {j}k_c \mu ^2}{8 z \Omega ^2}\left( {\bar{t}}_1^2-{\bar{t}}_2^2\right) \right] \exp \left[ -\frac{\text {j}k_c^3}{8 z^3 \Omega ^2}\left( x_1^2-x_2^2\right) \right] \nonumber \\& \times \exp \left[ -\frac{\left( \mu {\bar{t}}_1 - k_c x_2/z \right) ^2 + \left( \mu {\bar{t}}_2 - k_c x_1/z \right) ^2}{16W_x^2\Omega ^2} \right] \nonumber \\& \times \exp \left\{ -\frac{\left[ \left( \mu {\bar{t}}_1 - k_c x_2/z \right) - \left( \mu {\bar{t}}_2 - k_c x_1/z \right) \right] ^2}{8\delta _x^2\Omega ^2} \right\} \nonumber \\& \times \exp \left[ \text {j}\mu \frac{k_c^2}{4 z^2 \Omega ^2}\left( x_1 {\bar{t}}_2 - x_2 {\bar{t}}_1 \right) \right] , \end{aligned}$$

where $${\bar{t}}_{1,2} = t_{1,2} - z/c - x_{1,2}^2/\left( 2cz\right) 
$$,

14 $$\begin{aligned} \Omega ^2 = \left( \frac{1}{4W_x^2}\right) ^2\left( 1 + 4\gamma _x^2 + N_F^2\right) , \end{aligned}$$

and $N_F = 2 k_c W_x^2/z$ is the Fresnel number at the carrier frequency.

Like the spectral density above, the time-varying, ensemble-averaged intensity can be determined by evaluating the MCF at equal space and time points:

15 $$\begin{aligned} I\left( x,t,z\right)= & {} \Gamma \left( x,x,t,t,z\right) \nonumber \\= & {} \frac{N_F}{\sqrt{1+4\gamma _x^2+N_F^2}} \exp \left[ -\left( 1 + \mu ^2\frac{4W_x^2 W_t^2}{1+4\gamma _x^2+N_F^2}\right) \frac{{\bar{t}}^2}{2W_t^2}\right] \nonumber \\& \times \exp \left[ -\left( \frac{N_F^2}{1+4\gamma _x^2+N_F^2}\right) \frac{x^2}{2W_x^2}\right] \exp \left( \mu \frac{2 N_F}{1+4\gamma _x^2+N_F^2}{\bar{t}}x\right). \end{aligned}$$

The behavior of this beam in the *x*-*t* plane is the same as that described for the twisted space-frequency GSM source in the *x*-$\omega$ plane.

**Figure 1:**
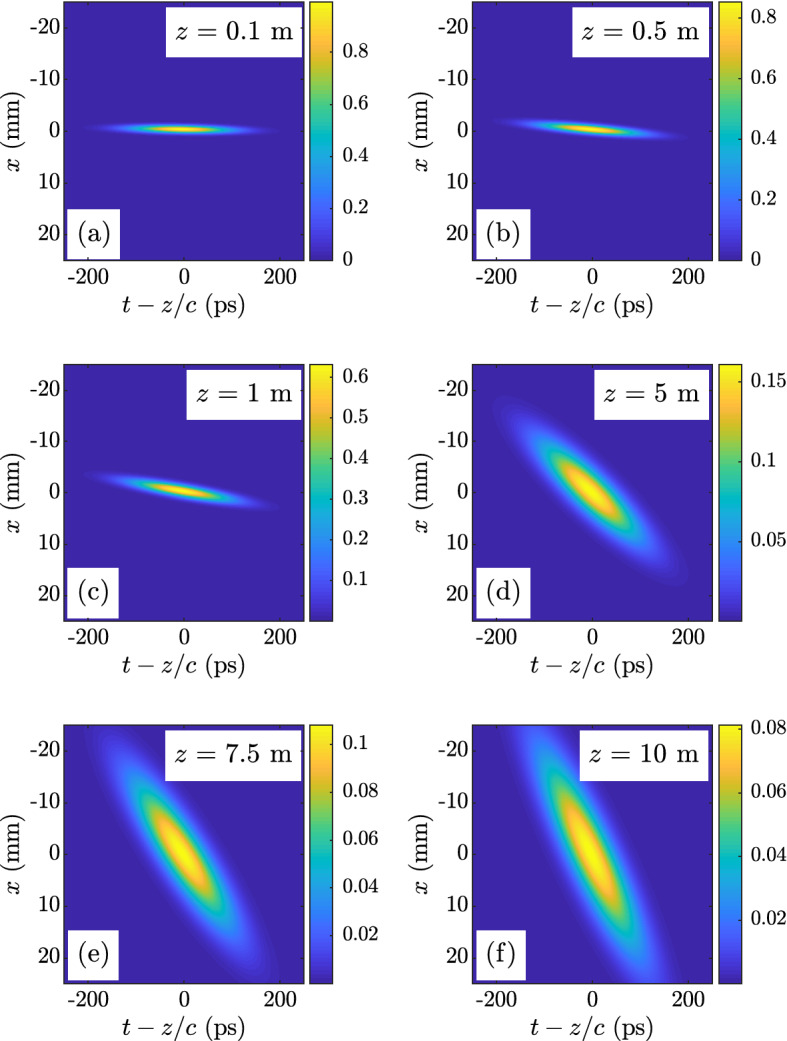
Mean intensity $I\left( x,t,z\right)$ for a twisted space-time GSM partially coherent source with $\lambda _c = 1 \text { } \mu \text {m}$, $W_x = 0.5 \text { mm}$, $\delta _x = 0.27 \text { mm}$, $W_t = 70.7 \text{ ps }$, $\delta _t = 33.3 \text { ps}$, and $\mu = 0.1 \text { } \left( \text {mm} \,\, \text {ps}\right) ^{-1}$—(**a**) $z = 0.1$ m, (**b**) $z = 0.5$ m, (**c**) $z = 1$ m, (**d**) $z = 5$ m, (**e**) $z = 7.5$ m, and (**f**) $z = 10$ m.

As briefly stated above, the time *t* paraxially corresponds to the physical propagation dimension $z$ (i.e., ${\bar{t}} \approx t - z/c$). As such, when $\mu \ne 0$, a twisted space-time beam tumbles as it propagates, and like beams with STOVs^23,25–27^, has a component of OAM in the $\pm y$ direction depending on the sign of $\mu$. Figure 1 and the corresponding Supplementary Video V1 show this behavior for an example twisted space-time GSM partially coherent beam.

## Results and discussion

In this section, we simulate the generation of twisted space-frequency and space-time GSM beams. First, we discuss the details of the wave-optics simulations.

### Simulation setup

For these simulations, we used computational grids that were 512 points per side. The grid spacings were approximately $\Delta x = 26.9$ $\mu$m and $\Delta \omega = 1.11$ GHz in the *x* and $\omega$ dimensions for the space-frequency source, and $\Delta x = 26.9$ $\mu$m and $\Delta t = 1.11$ ps in the *x* and *t* dimensions for the space-time source.

To generate twisted space-frequency and space-time GSM field realizations, we used their respective superposition rules in Eqs. (1) and (9) as detailed in Ref.^45^. For the paper’s completeness and the reader’s convenience, we briefly review this synthesis method using the space-time GSM source as an example.

A stochastic instance of a space-time GSM partially coherent source can be generated by evaluating the following integral:

16 $$\begin{aligned} U\left( x,t\right) = \iint _{-\infty }^{\infty } r\left( v_x,v_t\right) \left[ \frac{1}{2}p\left( v_x,v_t\right) \right] ^{1/2} H\left( x,t;v_x,v_t \right) \text {d}v_x\text {d}v_t, \end{aligned}$$

where *p* and *H* are given in Eq. (10) and *r* is a complex, delta-correlated, Gaussian-distributed stochastic function^45^. Taking the autocorrelation of Eq. (16) and noting that $\langle r\left( v_{x1},v_{t1}\right) r^*\left( v_{x2},v_{t2}\right) \rangle = 2 \delta \left( v_{x1}-v_{x2}, v_{t1}-v_{t2}\right)$ reproduces the space–time source superposition rule given in Eq. (9).

We now express Eq. (16) in discrete form as the integrals are evaluated numerically. In addition, *p* and *H* are separable in $v_x$ and $v_t$. Because of this, we can express the four-dimensional kernel *H* as the product of two three-dimensional kernels. This results in significant savings in computer memory. We therefore express Eq. (16) as the Hadamard product of two matrix-vector products:

17 $$\begin{aligned} \begin{gathered} {\varvec{U}}\left[ ij\right] = \underline{\mathbf {H}}\left[ ij,m\right] \left\{ {\varvec{r}}\left[ m\right] \odot \sqrt{{\varvec{p}}\left[ m\right] } \right\} \sqrt{ \frac{\Delta {v_x}}{2}} \odot \underline{\mathbf {H}}\left[ ij,n\right] \left\{ {\varvec{r}}\left[ n\right] \odot \sqrt{ {\varvec{p}}\left[ n\right] } \right\} \sqrt{ \frac{\Delta {v_t}}{2}} \end{gathered}, \end{aligned}$$

where *m*, *n* are the discrete $v_x,v_t$ indices, $\Delta {v_x},\Delta {v_t}$ are the spacings in the $v_x,v_t$ dimensions, and *ij* is a double index corresponding to every combination of discrete *x*, *t* coordinates.

The kernels $\underline{\mathbf {H}}$ are $\left( N_xN_t\right) \times N_{v_x}$ or $\left( N_xN_t\right) \times N_{v_t}$ matrices, where $N_x,N_t$ and $N_{v_x},N_{v_t}$ are the number of grid points in the *x*, *t* and $v_x,v_t$ dimensions. The ${\varvec{p}}$ and ${\varvec{r}}$ are $N_{v_x} \times 1$ or $N_{v_t} \times 1$ vectors, and ${\varvec{U}}$—the stochastic field realization—is an $\left( N_xN_t\right) \times 1$ vector, which must be reshaped to an $N_x \times N_t$ matrix. The ${\varvec{r}}$ are vectors of standard complex normal random numbers.

In these simulations, $N_x = N_t = N_{v_x} = N_{v_t} = 512$, $\Delta {v_x} = \left( N_x \Delta x\right) ^{-1} = 72.7$ m$^{-1}$, and $\Delta {v_t} = \left( N_t \Delta t\right) ^{-1} = 1.76$ GHz. Likewise, for the space-frequency source simulations, $N_x = N_\omega = N_{v_x} = N_{v_\omega } = 512$, $\Delta {v_x} = \left( N_x \Delta x\right) ^{-1} = 72.7$ m$^{-1}$, and $\Delta {v_\omega } = \left( N_\omega \Delta \omega \right) ^{-1} = 1.76$ ps. Table 1 lists the parameter values for the simulated twisted space-frequency and space-time GSM partially coherent beams.

**Table 1:** Simulated twisted space-frequency and space-time GSM beam parameters.

$\lambda _c$	1 $\mu$m
$W_x$	0.5 mm
$\delta _x$	0.269 mm
$W_\omega , W_t$	70.7 GHz, 70.7 ps
$\delta _\omega , \delta _t$	33.3 GHz, 33.3 ps
$\mu$	0.10 $\left( \text {mm} \, \text {GHz} \right) ^{-1}$, 0.10 $\left( \text {mm} \,\, \text {ps} \right) ^{-1}$
$\sigma _x$	11 mm$^{-2}$
$\sigma _\omega ,\sigma _t$	700 THz$^{-2}$, 700 ns$^{-2}$
$\alpha$	0.13 mm$^{2}$
$\beta$	0.002 THz$^{2}$; 0.002 ns$^{2}$

After generating a field instance using Eq. (17) and the values listed in Table 1, we digitally propagated *U* $z = 0.126~\text {m}$, 0.262 m, 0.524 m, 1.05 m, 3.14 m, and 6.28 m—corresponding to Fresnel numbers $N_F = 25$, 12, 6, 3, 1, and 0.5, respectively. For the twisted space-frequency field instances, we performed the propagations by evaluating the Fresnel integral along the *x* dimension of each realization *U* using a fast Fourier transform (FFT)^46,47^. The process was slightly different for the twisted space-time field realizations. We first transformed the twisted space-time field instance *U* to the *x*-$\omega$ domain using a FFT computed along the *t* dimension of *U*. We then propagated that field using the Fresnel integral (again, evaluated using a FFT) computed along the *x* dimension of *U*. Lastly, we transformed the field back to the *x*-*t* domain using a FFT computed along the $\omega$ dimension of *U*.

From 5,000 propagated field realizations of twisted space-frequency and space-time GSM partially coherent beams, we computed the sample spectral densities *S* and mean intensities *I*, respectively. In addition, we computed planar slices through the four-dimensional CSDs *W* and MCFs $\Gamma$, respectively.

In the next section, we compare these sample moments to their corresponding theoretical quantities derived and discussed earlier in the paper. The purpose of this is twofold: to verify that we have indeed produced the desired twisted space-frequency or space-time GSM source, and to validate our theory presented in the prior section.

We have included the MATLAB R2018b scripts (.m files) required to execute these wave-optics simulations as Supplementary Code C1.

### Results

**Figure 2:**
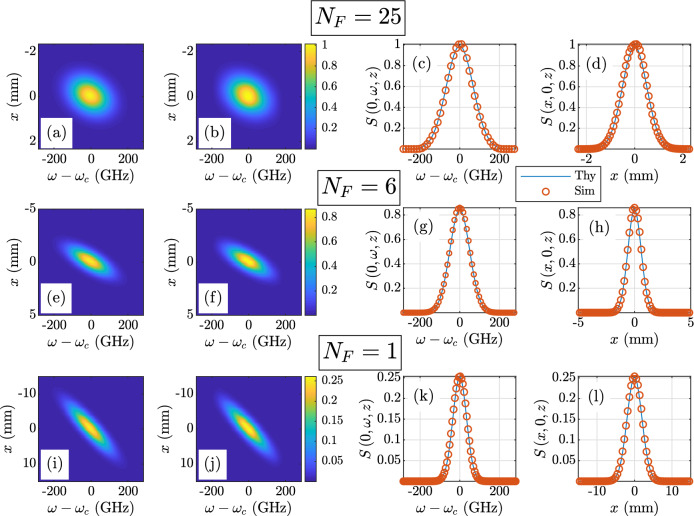
Twisted space-frequency GSM spectral density $S\left( x,\omega ,z\right)$ results. The theoretical spectral density $S^{\text {thy}}$ is given in Eq. (8): $N_F = 25$—(**a**) $S^{\text {thy}}$, (**b**) $S^{\text {sim}}$, (**c**) $S\left( 0,\omega ,z\right)$ theory versus simulation, and (**d**) $S\left( x,0,z\right)$ theory versus simulation; $N_F = 6$—(**e**) $S^{\text {thy}}$, (**f**) $S^{\text {sim}}$, (**g**) $S\left( 0,\omega ,z\right)$ theory versus simulation, and (**h**) $S\left( x,0,z\right)$ theory versus simulation; $N_F = 1$—(**i**) $S^{\text {thy}}$, (**j**) $S^{\text {sim}}$, (**k**) $S\left( 0,\omega ,z\right)$ theory versus simulation, and (**l**) $S\left( x,0,z\right)$ theory versus simulation.

**Figure 3:**
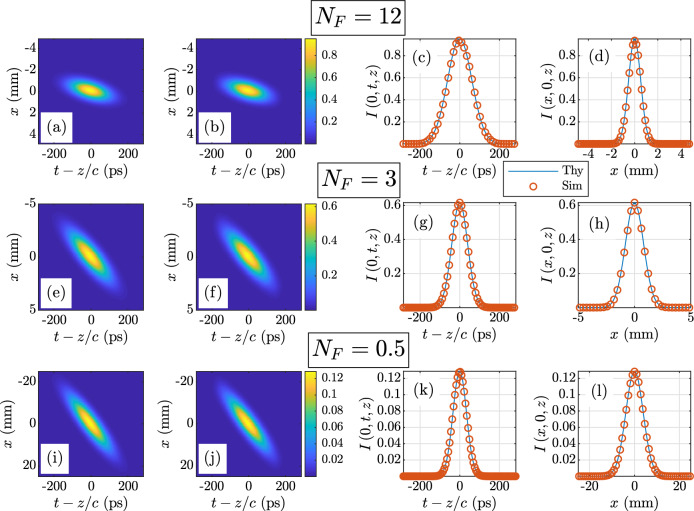
Twisted space-time GSM mean intensity $I\left( x,t,z\right)$ results. The theoretical mean intensity $I^{\text {thy}}$ is given in Eq. (15): $N_F = 12$—(**a**) $I^{\text {thy}}$, (**b**) $I^{\text {sim}}$, (**c**) $I\left( 0,t,z\right)$ theory versus simulation, and (**d**) $I\left( x,0,z\right)$ theory versus simulation; $N_F = 3$—(**e**) $I^{\text {thy}}$, (**f**) $I^{\text {sim}}$, (**g**) $I\left( 0,t,z\right)$ theory versus simulation, and (**h**) $I\left( x,0,z\right)$ theory versus simulation; $N_F = 0.5$—(**i**) $I^{\text {thy}}$, (**j**) $I^{\text {sim}}$, (**k**) $I\left( 0,t,z\right)$ theory versus simulation, and (**l**) $I\left( x,0,z\right)$ theory versus simulation.

**Figure 4:**
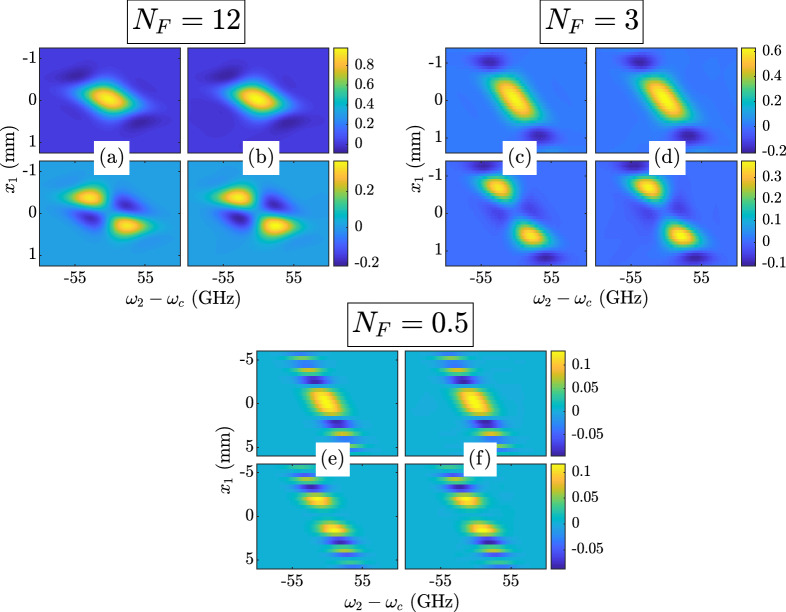
Twisted space-frequency GSM $W\left( x_1,0,\omega _c,\omega _2,z\right)$ results. The theoretical CSD $W^{\text {thy}}$ is given in Eq. (7): $N_F = 12$—(**a**) top ${\text {Re}}\left( W^{\text {thy}}\right)$, bottom ${\text {Im}}\left( W^{\text {thy}}\right)$ and (**b**) top ${\text {Re}}\left( W^{\text {sim}}\right)$, bottom ${\text {Im}}\left( W^{\text {sim}}\right)$; $N_F = 3$—(**c**) top ${\text {Re}}\left( W^{\text {thy}}\right)$, bottom ${\text {Im}}\left( W^{\text {thy}}\right)$ and (**d**) top ${\text {Re}}\left( W^{\text {sim}}\right)$, bottom ${\text {Im}}\left( W^{\text {sim}}\right)$; $N_F = 0.5$—(**e**) top ${\text {Re}}\left( W^{\text {thy}}\right)$, bottom ${\text {Im}}\left( W^{\text {thy}}\right)$ and (**f**) top ${\text {Re}}\left( W^{\text {sim}}\right)$, bottom ${\text {Im}}\left( W^{\text {sim}}\right)$.

**Figure 5:**
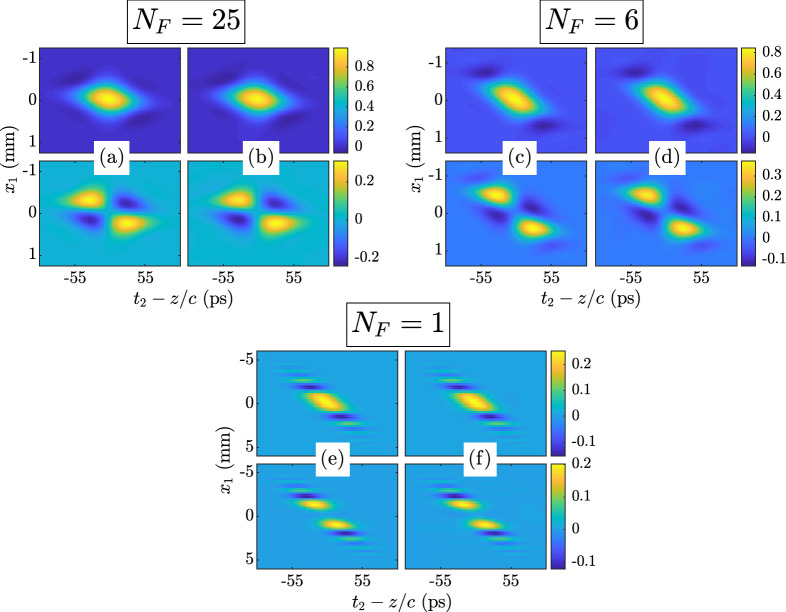
Twisted space-time GSM $\Gamma \left( x_1,0,z/c,t_2,z\right)$ results. The theoretical MCF $\Gamma ^{\text {thy}}$ is given in Eq. (13): $N_F = 25$—(**a**) top ${\text {Re}}\left( \Gamma ^{\text {thy}}\right)$, bottom ${\text {Im}}\left( \Gamma ^{\text {thy}}\right)$ and (**b**) top ${\text {Re}}\left( \Gamma ^{\text {sim}}\right)$, bottom ${\text {Im}}\left( \Gamma ^{\text {sim}}\right)$; $N_F = 6$—(**c**) top ${\text {Re}}\left( \Gamma ^{\text {thy}}\right)$, bottom ${\text {Im}}\left( \Gamma ^{\text {thy}}\right)$ and (**d**) top ${\text {Re}}\left( \Gamma ^{\text {sim}}\right)$, bottom ${\text {Im}}\left( \Gamma ^{\text {sim}}\right)$; $N_F = 1$—(**e**) top ${\text {Re}}\left( \Gamma ^{\text {thy}}\right)$, bottom ${\text {Im}}\left( \Gamma ^{\text {thy}}\right)$ and (**f**) top ${\text {Re}}\left( \Gamma ^{\text {sim}}\right)$, bottom ${\text {Im}}\left( \Gamma ^{\text {sim}}\right)$.

Because the behaviors of the twisted space-frequency and space-time GSM sources simulated here are identical in their respective domains (*x*-$\omega$ in the former, *x*-*t* in the latter), we present results for both that are complementary, such that the spectral density *S* and mean intensity *I*, and CSD *W* and MCF $\Gamma$ are presented for all $N_F$, but not duplicated.

We begin with the *S* and *I* results, which are shown in Figs. 2 and 3, respectively. The layout of the figures is identical. Proceeding down the rows, the *S* or *I* are displayed for a particular $N_F$. The Fresnel numbers are annotated on the figures for the reader’s convenience. Proceeding from left to right across the columns are the theoretical and simulated two-dimensional *S* or *I* in columns 1 and 2, respectively. The theoretical and simulated *S* or *I* images use the same false color scales defined by the color bars immediately following column 2. The last two columns report the one-dimensional profiles or slices through the theoretical and simulated (labeled “Thy” and “Sim” in the legends) *S* or *I*, plotted on the same axes for ease of comparison. Column 3 shows the $\omega$ or *t* slices, and column 4 displays the *x* slices.

The agreement between theory and simulation is excellent. The quality of these results imply that we have successfully generated twisted space-frequency and space-time sources that radiate the desired *S* and *I*. While these results are certainly positive, we still must verify that we have generated twisted space-frequency and space-time beams with the desired coherence properties. This requires examination of the cross-spectral density and mutual coherence functions, respectively.

Figures 4 and 5 show $W\left( x_1,0,\omega _c,\omega _2,z\right)$ and $\Gamma \left( x_1,0,z/c,t_2,z\right)$. We removed the piston phase shifts, $\exp \left[ \text {j}\left( k_1-k_2\right) z\right]$ and $\exp \left[ -\text {j}\omega _c\left( t_1-t_2\right) \right]$, from these results. Both figures consist of three groups of four images. Each image group corresponds to an $N_F$, labeled for the reader’s convenience. Inside each group, the four images are arranged in two rows and two columns. The first column presents the theoretical *W* or $\Gamma$, while the second column displays the simulated results. The first row shows the real parts of *W* or $\Gamma$; the second row shows the imaginary parts. The theoretical and simulated *W* or $\Gamma$ use the same false color scales defined by the color bars at rows’ end.

Again, the agreement between theory and simulation is excellent. The quality of these results, in combination with those in Figs. 2 and 3, prove that we have indeed generated twisted space-frequency and space-time GSM beams with the parameters given in Table 1.

### Physical synthesis

Before concluding, it is worth discussing how to physically synthesize these partially coherent beams. Here, we focus on twisted space-time GSM partially coherent sources, as the setup to synthesize twisted space-frequency GSM beams is similar.

**Figure 6:**
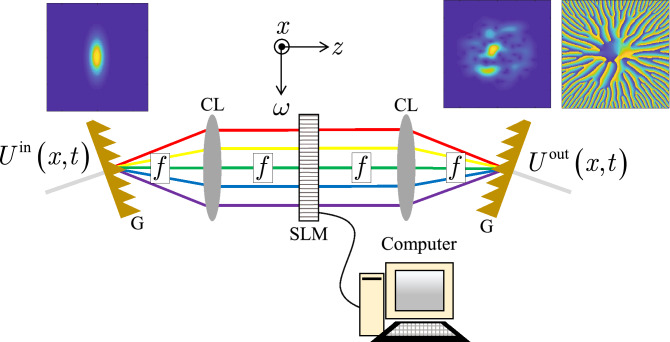
Fourier transform pulse shaper—G is grating, CL is cylindrical lens, and SLM is spatial light modulator.

Figure 6 shows a schematic of an optical system that can be used to synthesize a twisted space-time partially coherent source. This device is known as a Fourier transform pulse shaper^23,26,48–51^.

We assume a coherent Gaussian pulse as the input field into the shaper, i.e.,

18 $$\begin{aligned} U^{\text {in}}\left( x,t\right) = \exp \left( -\frac{x^2}{4W_x^2}\right) \exp \left( -\frac{t^2}{4W_t^2}\right) \exp \left( -\text {j}\omega _c t\right) . \end{aligned}$$

This field is incident on a grating (G), which in combination with a cylindrical lens (CL) of focal length *f*, maps the spectrum of $U^{\text {in}}$ into physical space at the plane of the spatial light modulator (SLM). The SLM modifies the field in the *x*-$\omega$ domain, introducing random space-frequency coupling. The field then transits another cylindrical lens (of focal length *f*) and an identical grating to the first. This combination reverses the spectrum-to-space mapping of the first G-CL system, resulting in a stochastic fully-coherent realization of a twisted space-time GSM partially coherent beam ($U^{\text {out}}$ in the figure). Partial coherence is introduced by incoherently summing many such realizations, or pulses. Although we have ignored it for mathematical convenience, we note that the spatial distribution of the beam in the *y* direction is generally unaffected by the pulse shaper^27^. The beam, therefore, can be spatially shaped either before or after the pulse shaper.

In contrast to the simulations and, in particular, Eqs. (16) and (17), the SLM in Fig. 6, which produces the field realization, operates in the *x*-$\omega$ plane. Therefore, the kernel *H* in Eq. (10) must be transformed into that domain, namely,

19 $$\begin{aligned} H\left( x,\omega ;v_x,v_t \right)= & {} \frac{1}{2\pi }\int _{-\infty }^{\infty } H\left( x,t;v_x,v_t \right) \exp \left( \text {j}\omega t\right) \text {d}t = H\left( x,\omega ;v_x \right) H\left( x,\omega ;v_t \right) \nonumber \\ H\left( x,\omega ;v_x \right)= & {} \sqrt{\frac{1}{2\sqrt{\pi \sigma _t}}} \exp \left( -\frac{\sigma _x}{2}x^2\right) \exp \left( -\frac{{\bar{\omega }}^2}{8\sigma _t}\right) \exp \left( \frac{\alpha ^2\mu ^2}{4\sigma _t}v_x^2\right) \exp \left[ -\text {j}\left( x + \frac{\alpha \mu }{2\sigma _t}{\bar{\omega }} \right) v_x \right] \nonumber \\ H\left( x,\omega ;v_t \right)= & {} \sqrt{\frac{1}{2\sqrt{\pi \sigma _t}}} \exp \left( -\frac{\sigma _x}{2}x^2\right) \exp \left( -\frac{{\bar{\omega }}^2}{8\sigma _t}\right) \exp \left( -\frac{v_t^2}{4\sigma _t}\right) \exp \left[ \left( \beta \mu x + \frac{1}{2\sigma _t}{\bar{\omega }} \right) v_t \right] . \end{aligned}$$

The coefficients for $H\left( x,\omega ;v_x\right)$ and $H\left( x,\omega ;v_t\right)$ are included for completeness. They scale the on-axis intensity of the beam and can be neglected. The kernel $H\left( x,\omega ;v_x\right)$ grows without bound because of the $v_x$ exponential; however, when multiplied by $\sqrt{p\left( v_x\right) }$, the superposition integral [see Eq. (16)] converges.

We close this section with a brief discussion of hardware considerations for the apparatus in Fig. 6. Note that additional information can be found in Refs.^23,26,48–51^ and the references cited therein. The most critical component in Fig. 6 is the SLM, which ideally, should cycle at the source’s pulse repetition frequency. This ensures that every pulse is statistically independent of every other pulse and therefore, quick convergence to the desired twisted space-time partially coherent beam.

SLM speed depends heavily on type, e.g., liquid-crystal SLMs have refresh rates of 100s of Hz, segmented deformable mirrors (DMs) and digital micromirror devices (DMDs) refresh at rates of 10s of kHz. Of course, speed is not the only consideration. Liquid-crystal SLMs can have millions of pixels—orders of magnitude more than segmented DMs—and are more light efficient than DMDs, which are binary devices. More information on these SLMs can be found in Refs.^52–59^. As mentioned in Ref.^51^, since the MCF (or CSD) is computed over the ensemble of all possible field, or pulse realizations, SLM speed does not matter if the goal is solely to produce the partially coherent source. Although obvious, SLM choice ultimately depends on the application.

## Conclusion

In this paper, we presented space-frequency and space-time extensions to Simon and Mukunda’s spatially twisted partially coherent beams. Like the recently introduced STOV fields, which provided the impetus for this work, twisted space-frequency and space-time partially coherent beams possess transverse OAM.

Starting with the superposition rule for genuine partially coherent sources, we derived the CSD and MCF for twisted space-frequency and space-time partially coherent sources, respectively. Assuming a GSM form for the twisted sources, we examined their free-space propagation behaviors by evaluating the paraxial CSD and MCF propagation integrals. We derived expressions for the spectral density and mean intensity for any plane $z > 0$ and described both physically.

To validate our work, we simulated the generation and propagation of example twisted space-frequency and space-time partially coherent beams. We described the details of our simulations and the stochastic field realization process. We compared the simulated, or sample second-order field moments—spectral densities, mean intensities, CSDs, and MCFs—to their corresponding theoretical expressions. The simulated and theoretical moments were found to be in excellent agreement.

Lastly, we described how to physically generate stochastic realizations of these beams using a device known as a Fourier transform pulse shaper, which consisted of two identical gratings, cylindrical lenses, and a SLM. We briefly discussed the characteristics of different types of SLMs, and the pros and cons of using them in the shaper to generate random pulse realizations.

Light that possesses transverse angular momentum is a relatively recent phenomenon and an exciting new area of beam control research. Considering the applications which use traditional, spatially twisted or vortex light^3–8,10–21^, we should expect that space-frequency or space-time twisted beams (including STOVs) will be used in optical tweezing, particle manipulation, optical communications, and astronomy in novel ways^24,26,60^. In addition, there has been recent work in coupling spin (concerns circular polarization) and orbital angular momenta resulting in novel light control, generation, and optical manipulation techniques^61–63^. Similar coupling is possible with space-frequency and space-time twisted partially coherent beams by generalizing the scalar analysis presented in this paper to include the vector or electromagnetic nature of these stochastic light sources. The work we present here adds to the exciting new field of light beams carrying transverse angular momentum as well as the existing, rich literature on partially coherent sources.

## Supplementary information

Supplementary Video 1.

Supplementary Information 1.

Supplementary Code C1.
