# Supplementary material for: Twisted space-frequency and space-time partially coherent beams
Source: Sci Rep. 2020 Jul 24;10:12443. doi: 10.1038/s41598-020-68705-9 (PMC7381620; doi:10.1038/s41598-020-68705-9)
Supplement: Supplementary file 2 — Supplementary Information 1. [file 41598_2020_68705_MOESM2_ESM.pdf]

Twisted space-frequency and space-time partially coherent beams: supplementary material

Milo W. Hyde IV[1,*]

[1]Air Force Institute of Technology, Dayton, OH 45433, USA
[*]milo.hyde@us.af.mil

Supplementary Video V1

Supplementary Video V1 is a 20 s animation which shows how a twisted space-time Gaussian Schell-model (GSM) beam evolves as it propagates. Figure S1 shows a single frame of that video. The video consists of four figures, all showing images or plots of the mean intensity in the x-t plane. The mean intensity of a twisted space-time GSM beam is

$$\begin{aligned} I(x,t,z) &= \Gamma(x,x,t,t,z) \\ &= \frac{N_F}{\sqrt{1+4\gamma_x^2+N_F^2}} \exp\left[-\left(1+\mu^2\frac{4W_x^2W_t^2}{1+4\gamma_x^2+N_F^2}\right)\frac{\bar{t}^2}{2W_t^2}\right] \exp\left[-\left(\frac{N_F^2}{1+4\gamma_x^2+N_F^2}\right)\frac{x^2}{2W_x^2}\right] \exp\left(\mu\frac{2N_F}{1+4\gamma_x^2+N_F^2}\bar{t}x\right), \end{aligned} \quad (1)$$

where $\bar{t} = t - z/c - x^2/(2cz)$, $\gamma_x^2 = W_x^2/\delta_x^2$, and $N_F = 2k_cW_x^2/z$ is the Fresnel number at the pulse's carrier frequency. The physical units for I are W/m.

The upper left image in Supplementary Video V1, i.e., (a), shows I for an untwisted, partially coherent GSM beam [$\mu = 0\ (\text{mm}\cdot\text{ps})^{-1}$]. Subimage (b) shows I for a $\mu = 0.1\ (\text{mm}\cdot\text{ps})^{-1}$ twisted GSM beam. Subplots (c) and (d) show the $x = 0$ and $t = z/c$ slices through the I in (a) and (b), respectively.

The untwisted GSM pulsed beam results are included to clearly show the effects of the twist parameter μ. Subplots (c) and (d) show how the pulse (temporal) and spatial beam widths change versus propagation distance z. For the untwisted beam, the pulse width does not depend on z, and the spatial beam width increases due to diffraction. In the case of the twisted space-time GSM beam, the pulse width decreases with increasing z; it does asymptote as $N_F \to 0$. Like the untwisted beam, the spatial beam width increases due to diffraction.

We note that the energies in both the untwisted and twisted GSM pulses are equal as physically expected due to the conservation of energy. This energy (in joules) is relatively straightforward to derive and proportional to

$$E \propto \iint_{-\infty}^{\infty} I(x,t,z)\,\mathrm{d}x\mathrm{d}t = 2\pi W_x W_t. \quad (2)$$

The fact that the spatial widths of both pulsed beams are equal, while the temporal width of the twisted GSM beam decreases with z, means that the twisted beam's intensity must be redistributed away from the x and t axes to keep the energy in both beams equal. This redistribution manifests as rotation.

We have included the MATLAB R2018b scripts (.m files) required to produce Supplementary Video V1 in Supplementary Code C1. The scripts are `Supplementary_Video_V1.m` and `Space_Time_Twist_MCF.m`.

Supplementary Code C1

`Supplementary_Code_C1.zip` is a ZIP compressed file archive containing the MATLAB R2018b scripts (.m files) necessary to run the simulations discussed in the main paper. It also contains the scripts required to produce Supplementary Video V1 discussed above.

`Supplementary_Code_C1.zip` contains the following files:

- `RUN_Twisted_Beams_Sim.m`—Executes `Space_Time_Twisted_Beams_Sim.m` and `Space_Frequency_Twisted_Beams_Sim.m`.

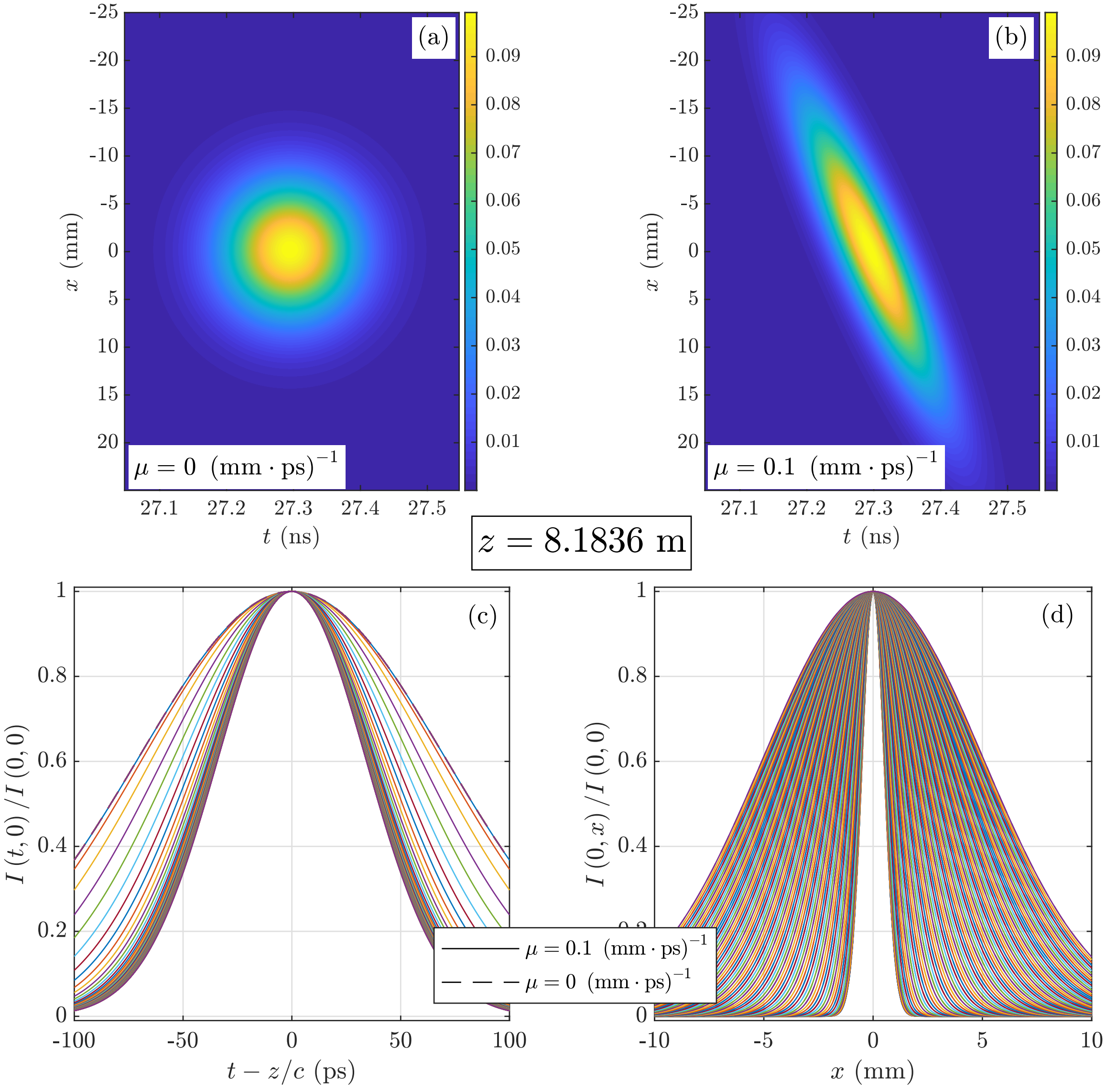

Supplementary Video V1: Mean intensity $I(x,t,z)$ for a twisted space-time GSM partially coherent source with $\lambda_c = 1\ \mu$m, $W_x = 0.5$ mm, $\delta_x = 0.27$ mm, $W_t = 70.7$ ps, and $\delta_t = 33.3$ ps—(a) $\mu = 0\ (\mathrm{mm}\cdot\mathrm{ps})^{-1}$, (b) $\mu = 0.1\ (\mathrm{mm}\cdot\mathrm{ps})^{-1}$, (c) normalized intensity $x = 0$ slices through (a) and (b), and (d) normalized intensity $t = z/c$ slices through (a) and (b).

Figure S 1. Example frame from Supplementary Video V1.

- `Space_Time_Twisted_Beams_Sim.m`—Executes twisted space-time GSM beam propagation simulations described in the main paper.
- `Space_Frequency_Twisted_Beams_Sim.m`—Executes twisted space-frequency GSM beam propagation simulations described in the main paper.
- `Space_Time_Twist_MCF.m`—Returns the theoretical twisted space-time GSM mutual coherence function (MCF) given twisted GSM beam parameters (W_x, δ_x, W_t, δ_t, and μ), x_1, x_2, t_1, t_2, and propagation distance z.
- `Space_Freq_Twist_CSD.m`—Returns the theoretical twisted space-frequency GSM cross-spectral density (CSD) function given twisted GSM beam parameters (W_x, δ_x, W_ω, δ_ω, and μ), x_1, x_2, ω_1, ω_2, and propagation distance z.
- `Fresnel_Prop.m`—Returns the paraxial optical field at any $z > 0$ by evaluating the Fresnel integral using a fast Fourier transform. The method takes as inputs a space-frequency field $U_i(\omega, x)$, spatial grid x_i at which U_i is evaluated, optical wavelength $\lambda = 2\pi c/\omega$, and propagation distance z.
- `Plot_Results.m`—Generates Figs. 2–5 in the main paper using the MATLAB formatted data files (.mat files) produced by `Space_Time_Twisted_Beams_Sim.m` and `Space_Frequency_Twisted_Beams_Sim.m`.
- `Supplementary_Video_V1.m`—Produces Supplementary Video V1 (`Supplementary_Video_V1.avi`) discussed above. In addition, it generates the example video frame image shown in Fig. S1.
